# The impact of geometry, intramural friction, and pressure on the antegrade longitudinal motion of the arterial wall: A phantom and finite element study

**DOI:** 10.14814/phy2.15746

**Published:** 2023-06-18

**Authors:** Sandra Sjöstrand, Alice Widerström, Ingrid Svensson, Patrick Segers, Tobias Erlöv, Åsa Rydén Ahlgren, Magnus Cinthio

**Affiliations:** ^1^ Department of Biomedical Engineering, Faculty of Engineering Lund University Lund Sweden; ^2^ IBiTech‐bioMMeda Ghent University Ghent Belgium; ^3^ Department of Translational Medicine Lund University Lund Sweden; ^4^ Department of Medical Imaging and Physiology, Skåne University Hospital Lund University Malmö Sweden

**Keywords:** artery, finite element modeling, longitudinal displacement, shear stress, ultrasound

## Abstract

Longitudinal motion of the carotid arterial wall, as measured with ultrasound, has shown promise as an indicator of vascular health. The underlying mechanisms are however not fully understood. We have found, in in vivo studies, that blood pressure has a strong relation to the antegrade longitudinal displacement in early systole. Further, we have identified that a tapered geometry and the intramural friction in‐between two parts of a vessel wall influence the longitudinal displacement. We therefore studied the interaction between pressure, vessel geometry and intramural friction, tapered and straight ultrasound phantoms in a paralleled hydraulic bench study and corresponding numerical models. Profound antegrade longitudinal motion was induced in the innermost part of both tapered phantoms and the numerical models, but to a lesser extent when intramural friction was increased in the simulations. Strong correlations (*R* = 0.82–0.96; *p* < 1e‐3; *k* = 9.3–14 μm/mmHg) between longitudinal displacement and pulse pressure were found in six of seven regions of interest in tapered phantoms. The motion of the straight phantom and the corresponding numerical model was smaller, on average zero or close to zero. This study demonstrates that tapering of the lumen, low intramural friction, and pressure might be important conducive features to the antegrade longitudinal motion of the arterial wall in vivo.

## 1 INTRODUCTION

**FIGURE 1:**
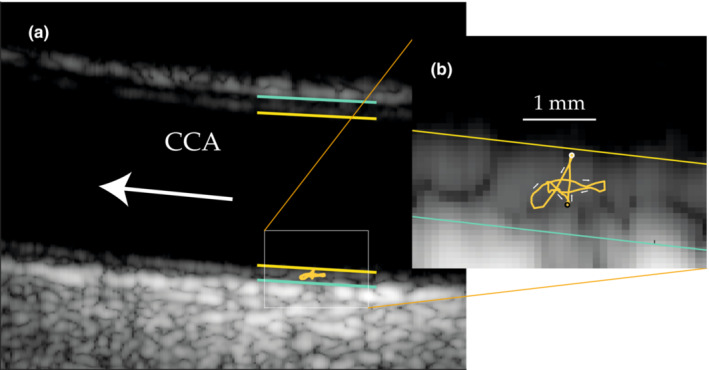
(a) Ultrasound image of the common carotid artery during a measurement of arterial wall motion of a 42‐year‐old female. The yellow lines mark the demarcations between lumen and intima and the green lines the demarcation between media and adventitia. The lines are 5 mm long. The white arrow marks the direction of the blood flow. (b) Enlargement of the far wall. The orange displacement vector curve shows the two‐dimensional wall motion during a cardiac cycle. The longitudinal motion of this vector curve is also shown in Figure 2b. The black circle marks the starting and ending positions as shown in Figure 2b, and the white circle marks the onset of the antegrade motion in early systole. The white arrows mark the direction of the motion. The white line marks 1 mm.

**FIGURE 2:**
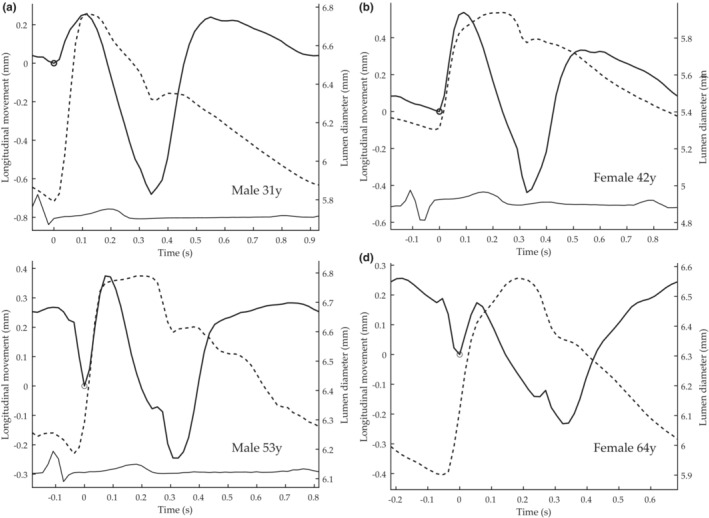
Longitudinal motion (solid) of the intima‐media complex of the far wall and the corresponding diameter change (dashed) in the common carotid artery of (a) a 31‐year‐old female, (b) a 42‐year‐old female, (c) a 53‐year‐old male, and (d) a 64‐year‐old female, after 10 min of rest. The subjects were healthy, non‐obese, non‐smoking and free from current medication. For longitudinal motion, a positive deflection denotes motion in the direction of blood flow. ECG is shown as reference (when available) at the bottom. The small circles mark the onset of an antegrade motion in early systole (also denoted by the origin of coordinates). The distinct antegrade motion is followed by a distinct retrograde motion in systole and a second distinct motion in early diastole. In the older subjects in (c) and (d), the first antegrade motion is preceded by a distinct retrograde motion. The longitudinal motion and diameter change were measured using the methods of Nilsson et al. (2013 and 2014), respectively. The curves shown are an average of curves from four to six cardiac cycles. Figures adapted from data collected from Cinthio et al. (Cinthio et al., 2018) and Ahlgren et al. (Ahlgren et al., 2023).

Hemodynamic forces acting on the arterial wall are considered important modulators of vascular tone and remodeling, and are increasingly recognized as playing an important role in atherogenesis (Nichols & O'Rourke, 2011). Fluid shear stress and lumen diameter change caused by pulsatile blood pressure have been subjects of extensive research (Nichols & O'Rourke, 2011) while the function and underlying mechanisms of longitudinal motion of the arterial wall remain largely unexplored. Distinct longitudinal motion (Cinthio et al., 2005; Golemati et al., 2003; Persson et al., 2002, 2003) is present in humans in both large muscular and elastic arteries (Cinthio et al., 2006). The displacement is largest in the part closest to the lumen, the intima‐media complex, and of the same magnitude as the diameter change (Figures 1 and 2, in vivo data from a related study). The outer part, the adventitia, shows the same basic pattern of motion but with smaller displacements, thereby demonstrating the presence of previously unknown substantial shear strain, and thus shear stress, intramurally (Cinthio et al., 2006; Idzenga et al., 2012; Nilsson et al., 2010; Zahnd, Boussel, Serusclat, & Vray, 2011). The most extensive shear strain seems to take place at, or near the interface between media and adventitia, the external elastic lamina (Cinthio et al., 2006; Nilsson et al., 2010).

Longitudinal motion of the common carotid artery in healthy humans at rest can show dramatically different multi‐phasic patterns, even in subjects of similar age and gender (Ahlgren, Cinthio, Persson, & Lindström, 2012; Cinthio et al., 2006; Yli‐Ollila et al., 2013). (Figure 2). We have shown that these patterns are stable over a 4‐month period (Ahlgren, Cinthio, Persson, & Lindström, 2012). The different patterns raise the question of whether the longitudinal motion might prove a valuable marker for future risk of cardiovascular disease (Ahlgren, Cinthio, Persson, & Lindström, 2012; Cinthio et al., 2006; Yli‐Ollila et al., 2013; Zahnd et al., 2015; Zahnd, Boussel, Marion, et al., 2011). That this might be the case is supported by recent studies (Taivainen et al., 2017, 2018, 2021). Furthermore, previous studies have reported that the amplitude of the longitudinal displacement of the arterial wall is reduced in patients with carotid plaques, suspected coronary artery disease, Type 2 diabetes (Svedlund et al., 2011; Svedlund & Gan, 2011; Zahnd, Boussel, Marion, et al., 2011), and periodontal disease (Zahnd et al., 2012). In addition, in a study on the porcine carotid artery, we reported that longitudinal motion and intramural shear strain undergo several hundred percent changes in response to the important endogenous hormones adrenaline and noradrenaline and that increase in longitudinal motion seems to be strongly related to α‐adrenoceptor activation (Ahlgren, Cinthio, Steen, et al., 2012). These findings might have important implications for vascular disease both in the short and long term, constituting a possible link between mental stress and cardiovascular disease (Ahlgren, Cinthio, Steen, et al., 2012). Furthermore, in studies on healthy humans we have recently shown that longitudinal motion and intramural shear strain undergo marked changes in response to exercise already at low workload (Ahlgren et al., 2023). A reasonable hypothesis is that these profound changes in longitudinal motion and intramural shearing can have a significant impact on the circulation of the vasa vasorum, endothelial function, smooth muscle cells, and the extracellular matrix components of the media, indicating the significance of longitudinal motion for vascular wall function (Ahlgren et al., 2023; Ahlgren, Cinthio, Steen, et al., 2012; Cinthio et al., 2006). However, in order to fully comprehend, the factors underlying longitudinal motion and intramural shearing are needed to be understood.

The multiphasic motion pattern implies to us that several, possibly opposing, active forces are involved. The intersubject variation of motion patterns (Ahlgren, Cinthio, Persson, & Lindström, 2012; Yli‐Ollila et al., 2013) (Figure 2), further suggests that the relative size and timing of the individual forces can vary. Active forces that have been suggested include shear stress from blood flow, ventricular contraction, blood pressure, and smooth muscles cell activation in the arterial wall (Ahlgren, Cinthio, Steen, et al., 2012; Athaide et al., 2022; Au et al., 2016; Cinthio et al., 2006; Idzenga et al., 2012; Yli‐Ollila et al., 2013; Zahnd et al., 2015). Specific phases of the longitudinal motion might also be associated with the diameter change and elastic recoil (Athaide et al., 2022; Cinthio et al., 2006; Fukui et al., 2007).

There are many indications that support that the ventricular contraction pulls down the proximal vasculature through the ventricular‐vascular coupling and cause the retrograde longitudinal motion in systole in the common carotid artery (Athaide et al., 2022; Au et al., 2016; Cinthio et al., 2006; Hao et al., 2021; Zahnd et al., 2015; Zhu et al., 2021). It is, however, more difficult to determine the underlying physiological mechanism driving the antegrade longitudinal motion as the shear stress from the flowing blood, the pulse blood pressure and the diameter change of the arterial wall are so intertwined.

A seemingly plausible hypothesis was that the shear stress from blood flow is an important factor underlying the antegrade longitudinal motion in early systole at the measurement site (Au et al., 2016; Cinthio et al., 2006; Hao et al., 2021). However, we found, in a pilot study on humans, that if isotropic elasticity is assumed the shear stress is minute in comparison to the longitudinal strain (Nilsson et al., 2009). In addition, in pharmacological experiments on the porcine carotid artery in vivo, we found that a profound increase in antegrade longitudinal displacement of the intima‐media complex can take place independently of wall shear stress from the blood flow (Ahlgren et al., 2015). This was clearly shown when a combination of adrenaline and noradrenaline was given during β‐blockade, thus, when cardiac contractility was reduced and flow velocity low (Ahlgren, Cinthio, Steen, et al., 2012). This is also supported by a case study using premature ventricular contractions to study the carotid artery longitudinal wall motion (Stevens & Au, 2021). In that study the antegrade longitudinal displacement in early systole remained unchanged despite large deviations in local blood velocity. Furthermore, we did not find any relationship between wall shear stress and the antegrade longitudinal displacement in early systole when studying arterial wall motion in healthy humans during submaximal physical activity (Ahlgren et al., 2023).

There is, on the other hand, circumstantial evidence suggesting that blood pressure can influence the antegrade longitudinal motion directly. (1) The timing of the antegrade phase of motion in early systole in relation to the diameter change shows good agreement with the expected arrival of the pulse wave (Ahlgren, Cinthio, Steen, et al., 2012; Athaide et al., 2022; Cinthio et al., 2006, 2018). (2) In a pharmacological study on the porcine carotid artery in vivo, creating a wide range of pulse pressures, peripheral vascular resistances, and blood flows, we found a strong correlation (*R* = 0.72, *p* < 0.001) between the antegrade longitudinal displacement and pulse pressure (Ahlgren, Cinthio, Steen, et al., 2012). (3) When studying arterial wall motion on healthy humans during submaximal physical activity we found that 25% of the subjects had a very strong or extremely strong correlation (0.8 < *R* ≤ 1, *p* < 0.001) between the antegrade displacement in early systole and systolic blood pressure (Ahlgren et al., 2023). However, so far, no plausible mechanism how the pulse pressure can directly actuate an antegrade displacement has been shown.

It has been suggested that pulse pressure can influence the longitudinal motion indirectly via the diameter change through a longitudinal‐circumferential mechanical coupling and helical oriented fibers (Athaide et al., 2022). Such a coupling has been incorporated in numerical models (Bukač et al., 2013; Fukui et al., 2007; Hodis & Zamir, 2009, 2011; Warriner et al., 2008). However, in these studies the antegrade longitudinal motion does not start at the same time as the diameter change starts to increase, as seen in healthy humans, that is, there is a phase difference between the diameter change curve and the longitudinal motion curve that is not present in early systole in vivo. This type of “out‐of‐phase” longitudinal motion has also been shown in straight tubular phantoms suspended in water (Salles et al., 2015). In our opinion, a passive longitudinal‐circumferential mechanical coupling cannot explain the antegrade longitudinal displacement in early systole. This is supported by the fact that the antegrade displacement increased up to 196% in the pharmacological study on the porcine carotid artery in vivo whereas the diameter change only increased 33% (Ahlgren, Cinthio, Steen, et al., 2012). Further, in the study on the common carotid artery of healthy humans during submaximal physical activity the antegrade displacement increased by mean 2565% when the diameter change only increased by mean 44% (Ahlgren et al., 2023).

In a computational model study, Bukač and Čanić showed that the geometry of the arteries are of importance for the longitudinal motion of the arterial wall (Bukač & Čanić, 2013). Recently, we identified that a phantom of a vessel incorporating a tapered region and low intramural friction in‐between the two parts of the wall can exhibit a distinct antegrade longitudinal motion, independent of that caused by the distension, both upstream and downstream of the tapered region (Sjöstrand et al., 2017). Therefore, to investigate whether the antegrade longitudinal motion seen in early systole, when the lumen diameter starts to increase, can be driven directly by the blood pressure, the aim of this study was to investigate the relation between longitudinal displacement, radial displacement, tapering, intramural friction, and pressure in four two‐part phantoms with different taper angles, subjected to various pulsatile flow and pressure. Corresponding finite element models were used to distinguish the effects of pressure from those of shear force, study the motion throughout the volume, and to vary friction between the two parts.

## 2 MATERIALS AND METHODS

### 2.1 Phantom experiments

**FIGURE 3:**
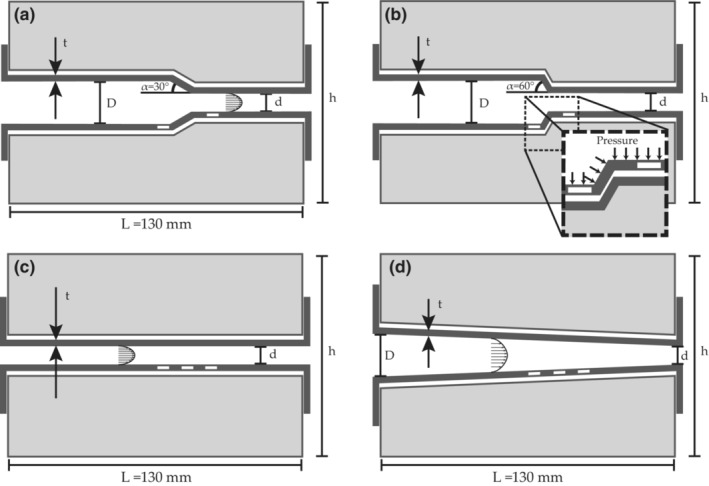
Schematic, cross‐sectional view along the centre axis of the four phantoms produced. *L* = 130 mm, *h* = 70 mm, *t* = 2 mm, *D* = 16 mm, *d* = 6 mm. These dimensions were from the unloaded phantoms; L refers to the length of the surrounding block; the inner part was 10 mm longer to make assembly possible. The figure is not to scale. (a) A phantom containing a tapered region with an inclination angle of 30°, (b) with a taper angle of 60°, (c) a straight phantom, and (d) a continuously tapered phantom with a taper angle of 2°. The flow was as indicated from left to right in the figures, and the distance from inlet to the beginning of the tapered region in (a) and (b) was 70 mm. In the enlarged section of sub section (b), pressure is indicated as vectors perpendicular to the surface. Upon assembly, the inner part inflated and filled the one‐millimeter gap shown between the parts in the figure. The measurement positions in the experiment are marked by white rectangles.

**FIGURE 4:**
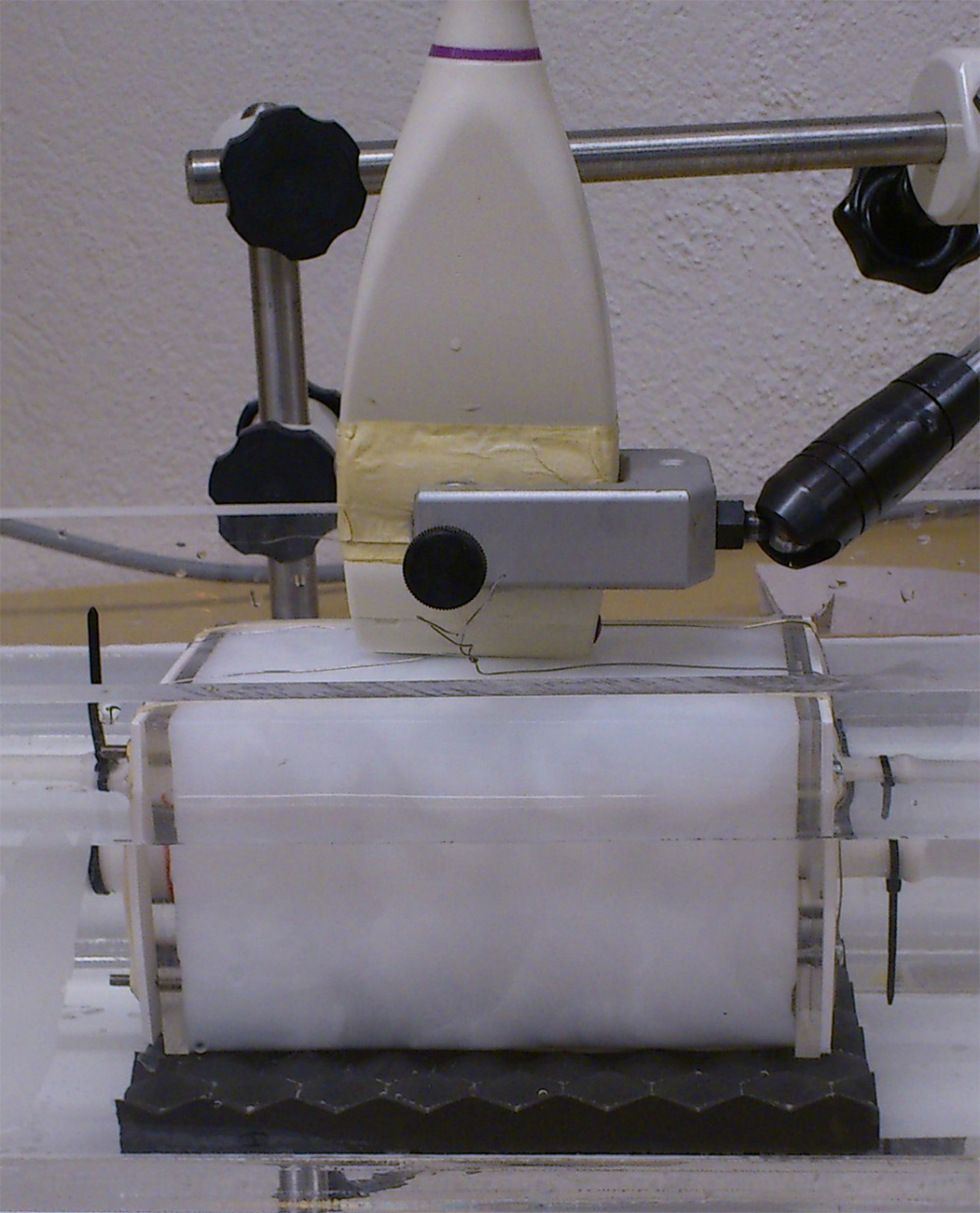
Photograph of the working phantom submerged in water. The direction of flow is from left to right in the image. The acoustic dampening material (black) can be seen beneath the phantom and above it the transducer and clamp.

Wall motion in four ultrasound phantoms was characterized in response to dynamic pressures in a paralleled hydraulic bench study. Polyvinyl alcohol cryogel (PVA‐C) phantoms were fabricated in two parts (Figure 3) to allow sliding of the inner wall in respect to the outer (Sjöstrand et al., 2017), emulating a mechanical interface between the media and adventitia of the arterial wall. Two of the phantoms had a tapered region (taper angles 30° and 60°) at the center, where the diameter narrowed from 16 mm to 6 mm; one had a constant lumen diameter of 6 mm, and one was continuously tapered by 2° (Figure 3). The inner parts were manufactured with brims at both ends used for connections (Widman et al., 2015), and the walls were 2 mm thick. Four outer parts were cast to enclose each of the inner parts. Finally, the assembled phantoms (one e.g., in Figure 4) were secured with wires to maintain the initial length throughout the pressure cycles.

**FIGURE 5:**
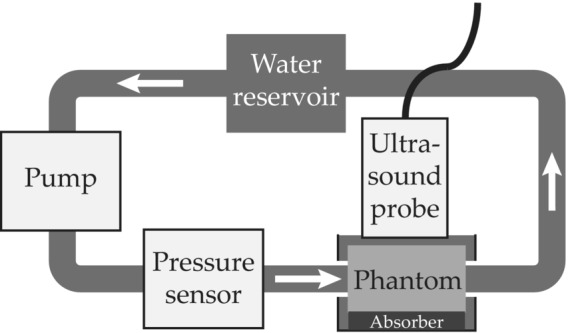
Diagram of the experimental setup. The phantom was placed on a sound‐absorbing material and connected to the pump and reservoir via elastic tubes. The pump and pressure sensor were computer‐controlled. The ultrasound equipment stored cine loops of B‐mode images, and post processing to extract motion data was performed offline.

A water layer in between the two walls allowed penetration of the ultrasound and reduced intramural friction. The assembly and measurements of the phantoms were, therefore, conducted in a water tank. The phantom was connected via tubes to a pump system (Figure 5) (Eriksson et al., 2000).

To measure the pressure, a sensor (Keller AG, Winterthur, Switzerland) was placed between the pump and the phantom (Figure 5). The pump output was controlled through the software Mint (ABB, Zürich, Switzerland). Five settings were prepared that gave pulsatile pressures with a frequency of 0.6 Hz and different pulse pressures, ranging from 20 to 45 mmHg for the tapered phantoms and 40–80 mmHg for the straight, due to the difference in flow resistance. The created pulsatile flow was tilted triangle shaped where the acceleration was five times faster than the deacceleration. For every pump setting, three measurements were performed on each phantom. Each measurement consisted of five consecutive pressure cycles.

To measure the motion induced in a phantom, an ultrasound system, HDI 5000 with the transducer L12‐5 (Philips Medical Systems), was used. The transducer was secured using a clamp on a jointed arm. Each ultrasound cine loop was manually terminated simultaneously with the sensor recording in order to allow synchronization. The system returned to its starting position before the next measurement. The ultrasound cine loops, along with pressure profiles, were analyzed using an in‐house developed software. The software estimated the mean pulse pressure, distension (Nilsson et al., 2014), and longitudinal displacement (Albinsson et al., 2014) of four pressure cycles in each sequence.

Two or three distinct echoes in the far wall were selected to be tracked and used in each of the ultrasound sequences. The tracked echoes were situated at approximately the same distance from the outlet in the different phantoms, 10 mm distal and 5 mm proximal of the tapering and, where applicable, in‐between (white boxes in Figure 3). The same procedure was carried out for all phantoms.

### 2.2 Simulations

To obtain an overview of the wall motion, numerical models of the phantoms were analyzed using the finite element analysis software Abaqus (Dassaults Systèmes, Vélizy‐Villacoublay, France; version 6–14.1). In these models we tested whether an increase in the internal pressure in the absence of flow can drive a longitudinal wall motion. The qualitative influence of the interaction between wall layers was also explored and was modeled as intramural friction.

An implicit solver was used to impose a uniform internal pressure during a general static step. With nonlinear geometric effects enabled, the solution describing the resulting geometry after perturbation by an internal pressure was incrementally obtained, using software‐controlled increments of 10^−5^ to 0.1.

**FIGURE 6:**
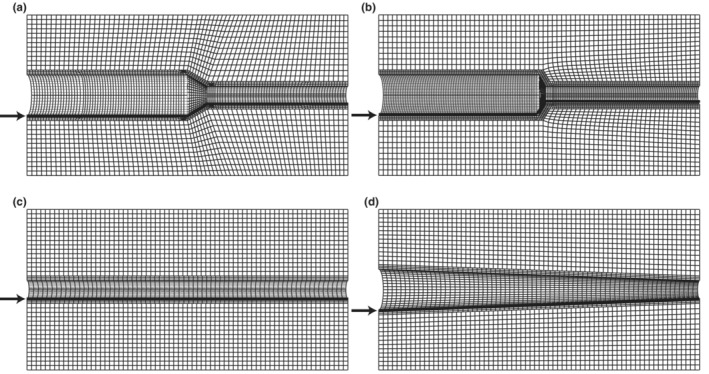
Finite element meshes of (a) the 30°, (b) 60°, (c) straight (d) continuously 2°narrowing models. The inner part consists of approximately 20,000 elements, the surrounding cylinder contains approximately 30,000, making a total of 50,000 elements for the complete model. The inner part was comprised of three layers of elements. Displacements were extracted from the nodes indicated by thicker black lines and arrows.

Four geometries, corresponding to the phantoms, were used in the simulations (Figure 6). Each consisted of an inner part with a wall thickness of 2 mm and a length of 140 mm inside a cylinder with a radius of 35 mm. The two parts were positioned in direct contact, the smallest diameter was 8 mm and the tapered lumen diameters decreased from 18 mm to match the dimensions of the mounted phantoms under static pressure from the water reservoir.

The geometries were spatially discretized using structured meshes consisting of linear, eight‐node hexahedral elements and swept meshing algorithms. A mesh dependency study (data not shown) demonstrated that, for the model with constant diameter, a model consisting of at least two layers of elements and 500 elements in total for the inner part converged in terms of longitudinal displacement, radial distension, and wall compression. In the subsequent models the mesh was locally refined in the tapered regions and structured by partitioning and edge seeding, ensuring mesh quality with aspect ratios smaller than 5. In these geometries, the converged mesh of the inner part of the vessel wall consisted of three layers of elements (over 20,000 in total). To mimic the outer part of the vessel wall, over 30,000 elements, of the same type, were used for the surrounding cylinders.

A contact definition in Abaqus connected the inner wall and the surrounding cylinder. The inner surface of the surrounding part was defined as master and the outer surface of the inner part as slave. The tangential force was defined as frictionless except when the influence of intramural friction was specifically studied. The friction coefficient was set to a constant in the range 0 ≤ *μ* ≤ 0.06, to simulate low intramural friction as observed in the phantoms. The normal force was defined as hard contact. The boundary conditions at the inlet and outlet planes of the models were zero displacement of the nodes in all three directions to match the boundary conditions in the experiment. The material was defined as isotropic and linearly elastic. An estimation of Young's modulus was achieved by fitting the computed radial displacements to our experimental results for the phantom with a constant radius. An internal pressure used in the experiment, 48 mmHg, was applied to all inner wall nodes in the numerical model and the displacement was compared to experimental results at the positions indicated in Figure 3c. This was repeated with the value of the Young's modulus varied in the range 30–80 kPa. A good match was obtained for 32 kPa, and this was used in the following simulations. The Poisson's ratio was set to 0.45, adapted from the literature (Fromageau et al., 2003, 2007). The same material properties were used for the inner and outer parts.

The effect of intramural friction on the longitudinal motion was investigated on one of the tapered models, the 60° model. A low pressure from the experiments (24 mmHg) was applied, and the response was compared for six cases (coefficients of friction 0, 0.001, 0.01, 0.02, 0.04, and 0.06). The displacements were extracted from nodes along the innermost surface (bold line in Figure 6). The intramural friction (coefficients of friction) was then set to zero, and each geometry was subjected to three different internal pressures (24, 34, and 44 mmHg). The pressures were chosen within the span of pulse pressures used in the experiments. The phantoms and models, along with corresponding pulse pressure ranges and simulated pressures, are summarized in Table 1.

**TABLE 1:** Summary of experimental and simulated conditions. The intramural friction coefficient was zero and the Young's modulus 32 kPa unless otherwise stated.

Geometry	Phantom pulse pressure range (mmHg)	Numerical models pressure (mmHg)
30° tapered region	25–45	24, 34, 44
60° tapered region	22–40	24, 34, 44
Straight	43–79	24, 34, 44
2° continuously tapered	25–39	24, 34, 44
Straight elasticity range 30–80 kPa	–	48
60° tapered region Intramural friction coefficients 0, 0.001, 0.01, 0.02, 0.04, 0.06	–	24

### 2.3 Statistics

Least squares regression analysis with calculation of Pearson's product moment correlation coefficient (*R*) was used to evaluate relations between longitudinal and radial displacements and pulse pressures. The *F*‐statistics was used to test the linear regression models (McClave & Sincich, 2003). *p* < 0.001 was considered significant.

## 3 RESULTS

### 3.1 Phantom experiments

**FIGURE 7:**
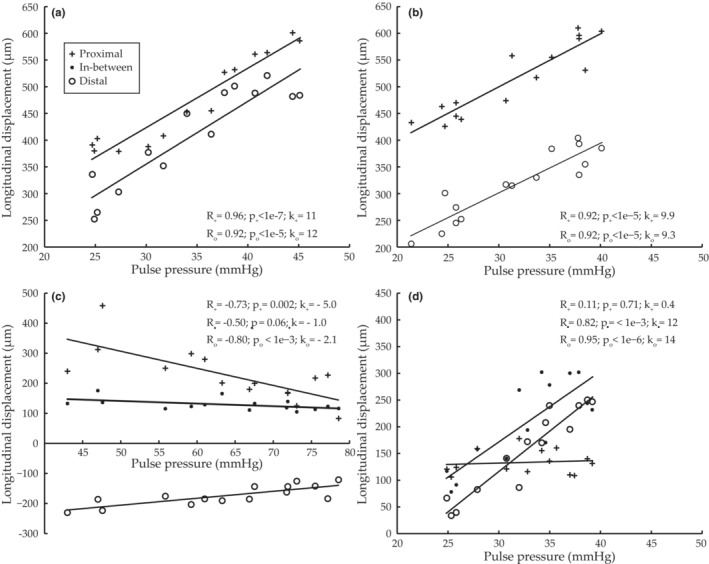
Results from phantom measurements of longitudinal wall displacements in relation to pulse pressure for the phantom with (a) 30° tapered region, (b) 60° tapered region, (c) straight lumen, and (d) continuously 2° narrowing lumen. Results extracted from the proximal position is marked with +, in‐between with •, and the distal position with o; see Figure 3 for measurement locations. Correlation coefficients *R* and *p* values, as well as the slope k (um/mmHg) are given. In the 2° continuously tapered phantom (d), the size of the distinct ultrasound echoes altered, especially in the proximal location, indicating out‐of‐plane motion and, thus, suboptimal measurements conditions.

A distinct forward, or antegrade, longitudinal displacement of the phantom wall could be observed for all tapered phantoms (Figure 7). The longitudinal displacements proximal and distal of the tapered region in the 30° and 60° phantoms showed positive correlation with pulse pressure (*R* = 0.92–0.96, *p* < 1e‐5, *k* = 9.3–12 μm/mmHg) (Figure 7a,b). Strong correlations (*R* = 0.82–0.95, *p* < 0.001, *k* = 12–14 μm/mmHg) were also found at two locations in the 2° continuously tapered phantom, but not in the proximal location (Figure 7d). The direction of the longitudinal motion of the straight phantom depended on location, and the displacement was negatively, and to a lesser extent, correlated with pulse pressure (*R* = −0.80 to −0.50, *p* = 0.0004 to 0.06; k = −5.0 to −1.0 μm/mmHg) (Figure 7c). All measured results were in‐line with the global trends observed visually. In the 2° continuously tapered phantom, the size of the distinct echoes altered, especially in the proximal location, indicating out‐of‐plane motion and, thus, suboptimal measurements conditions.

The correlation between radial distension and pulse pressure was significant for all phantoms, with *R* > 0.91 and *p* < 1e‐5.

### 3.2 Simulations

**FIGURE 8:**
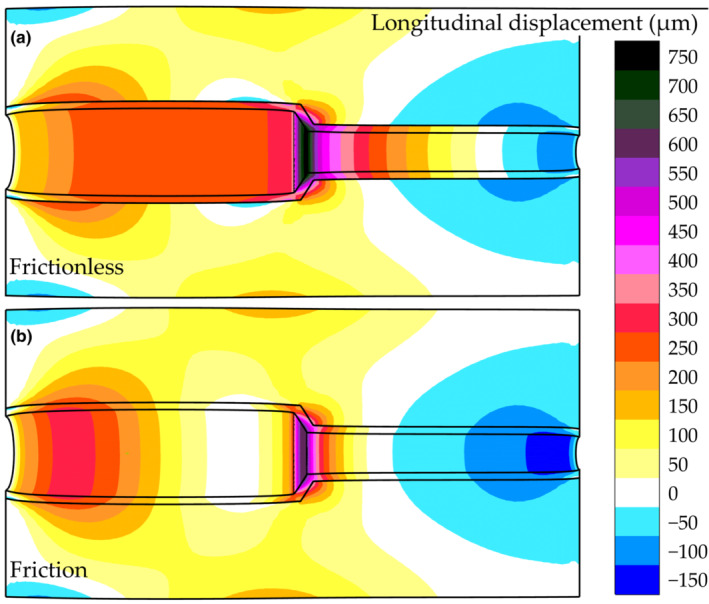
Contour plots of longitudinal displacement in 60° digital simulation model at 24 mmHg. (a) Represents the frictionless case and in (b) the coefficient of friction was 0.06. The scale is indicated to the right.

**FIGURE 9:**
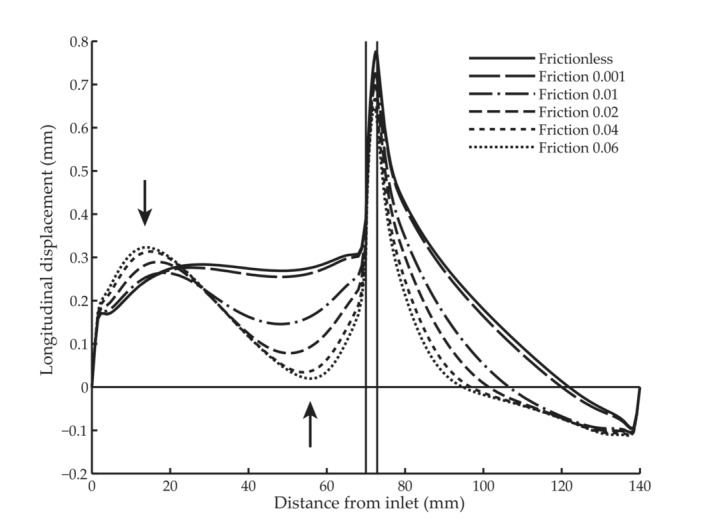
Longitudinal displacement as a function of distance from the inlet of the 60° digital simulation model with different coefficients of friction at the interface between the two parts. The arrows indicate the position of two local extrema proximal to the tapered region and the vertical lines show the tapered region.

Two contour plots illustrate how the inner and outer parts displace longitudinally in the 60° model at 24 mmHg pulse pressure for the lowest and highest intramural friction cases (Figure 8). The profile of the longitudinal wall displacement differed depending on the friction coefficient (Figure 9). The low intramural friction cases experienced an almost uniform longitudinal displacement proximal to the tapered region; for increased intramural friction, this region contained two clear local extrema (Figure 9). The narrow part of the phantom experienced an antegrade displacement close to the tapering and a retrograde displacement close to the outlet. A higher friction coefficient resulted in a larger retrograde region.

**FIGURE 10:**
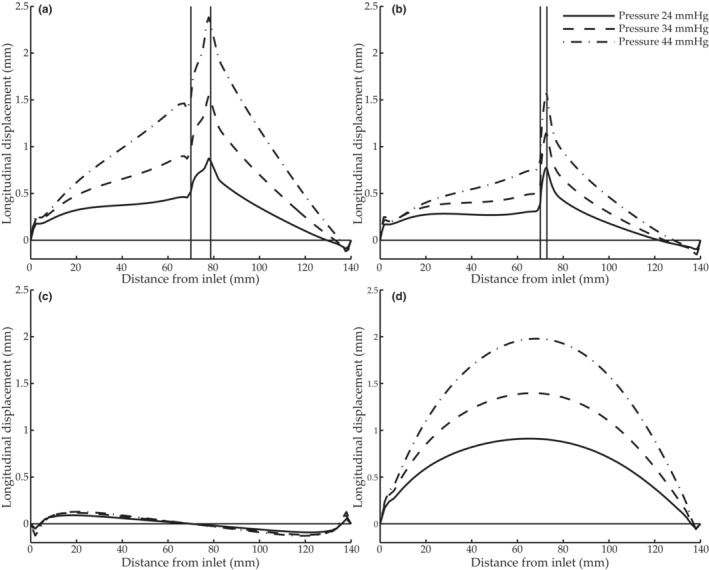
Longitudinal displacements of the digital simulation models with a (a) 30° tapered region, (b) 60° tapered region, (c) straight lumen, and (d) 2° continuously tapered lumen as a function of distance from inlet. The vertical lines show the beginning and end of the tapered regions. Each model was subjected to three different pressures, indicated in the legend in subfigure (b).

In locations corresponding to those analyzed in the phantoms (Figure 3), the simulations showed a positive relation between pressure and longitudinal displacement (Figure 10). When considering the motion profile over the entire length, it is clear that there was primarily antegrade motion in the tapered phantoms (Figure 10a,b,d), comparable in magnitude despite the range of taper angles. The longitudinal displacement of the straight model depended of location (Figure 10c) and was small, which was also the case in the experimental study (Figure 7c).

## 4 DISCUSSION

We have, for the first time, investigated the relationship between longitudinal displacement, intramural friction, and pressure in tapered phantoms and in silico models. The results showed that pressure can induce a longitudinal motion that is qualitatively different in tapered and straight geometries (Figures 7 and 10). The tapered models showed antegrade displacement throughout most of the model, whereas the displacement of the straight model was smaller and on average zero. There was a positive correlation between pulse pressure and longitudinal wall displacement both proximal and distal to a tapered region (Figure 7a,b). Such a correlation has previously been shown in porcine in vivo experiments (Ahlgren, Cinthio, Steen, et al., 2012), and when studying arterial wall motion in healthy humans during submaximal physical activity (Ahlgren et al., 2023). The same type of response was present in the simulations (Figure 10a,b) where the force was undoubtedly pressure as no other force was incorporated. In all tapered cases, regional and continuous, both phantoms and simulations, the extent of the longitudinal displacement increased with increasing pulse pressure (Figures 7a,b,d and 10a,b,d). Curiously enough, the relationship between the longitudinal motion and the pulse pressure in the three tapered phantoms (range 9–14 μm/mmHg) is almost identical to that found in the porcine common carotid artery in vivo (range 8–13 μm/mmHg) (Ahlgren, Cinthio, Steen, et al., 2012). Furthermore, the relationship between the longitudinal motion and pulse pressure is of the same magnitude as seen in those healthy humans which during exercise showed the strongest correlation between longitudinal displacement and systolic pressure (range 3–5 μm/mmHg) (Ahlgren et al., 2023).

The taper angles in this study may appear exaggerated or pathological; however, large angles are present at bifurcations in the arterial tree and small taper angles are seen in gradually narrowing arteries. Additionally, a small angle is also formed by the distension caused by the propagating pulse wave. The angle is less than 0.1° (if pulse wave velocity of 5 m/s, lumen diameter of 6 mm and a distension of 15% are assumed) but we suspect that its presence throughout the arterial tree might make it significant. The results from the 2° continuously tapered phantom and model showed that smaller angles can also be conducive to antegrade motion (Figures 7d and 10d). Undeniably, stenoses constitute the most significant case of narrowing in vivo. Such geometries would be highly conducive to antegrade longitudinal motion, but physiological changes, such as increased stiffness, might reduce overall longitudinal motion in pathological cases. Further studies of the interplay of factors affecting the longitudinal motion in vivo are needed to advance the discussion about the effect of stenosis on the longitudinal arterial wall motion.

In straight models, pressure can only act radially, and thus the longitudinal motion is intrinsically different. The distension of the wall causes the vessel to elongate, and the material is pulled toward the center, resulting in antegrade longitudinal displacement at the proximal end and retrograde distally when the internal pressure is increased (Figure 10c). This longitudinal‐circumferential mechanical coupling has previously been shown in mathematical models (Fukui et al., 2007; Warriner et al., 2008) and in phantoms (Salles et al., 2015). This elongation in longitudinal direction both within the straight phantom and model illustrates how the pressure acting radially can induce a longitudinal motion a distance from where the diameter change takes place (Figures 7c and 10c). Probably, this also happen in vivo distally to the pressure‐induced diameter change, manifested by a retrograde motion at late diastole as shown in Figure 2c,d. Two clear local extrema show up proximal to the tapered region when the intramural friction increased (Figure 9). We hypothesize these local extrema arise from the longitudinal‐circumferential mechanical coupling. However, further studies are needed to elucidate that.

Furthermore, we suggest that intramural friction between the arterial wall layers plays an important role for the longitudinal motion of the arterial wall. The shearing between media and adventitia (Cinthio et al., 2006; Idzenga et al., 2012; Nilsson et al., 2010; Zahnd, Boussel, Serusclat, & Vray, 2011) suggests that the layers are not rigidly connected. Previous simulation studies have established that fully tethered is an inadequate boundary condition for the outer arterial wall (Hodis & Zamir, 2009), and we suggest that this is the case intramurally as well. Thus, the interaction was modeled by low frictional sliding in our model. The longitudinal displacement of the frictionless inner part (Figure 8a) was antegrade for most of the length and generally greater than that of the surrounding part. When intramural friction was increased (Figure 8b), the displacements at the interface were more similar for the two parts. Furthermore, when the antegrade wall motion was inhibited, and a stationary region appeared proximal to the tapering (Figures 8b and 9). Qualitatively, the profile of the low friction cases corresponded well to observations from the phantom experiments.

The simplicity of our models entailed convenient handling of the phantoms and a clear interpretation of the results; the reductionistic models show that it is possible for the blood pressure to create an antegrade longitudinal wall motion under the specified conditions. However, given our results from pharmacological experiments in vivo (Ahlgren, Cinthio, Steen, et al., 2012), the possibility that smooth muscle activation and/or reorientation at the site of measurement may influence, or contribute to, the longitudinal motion of the arterial wall, must also be kept in mind.

Blood flow velocity and shear stress between the water and the phantom wall were not considered in this study, as several studies have shown that shear stress from the flowing blood does not seem to be an important conducive feature to antegrade longitudinal motion in vivo (Ahlgren et al., 2015, 2023; Nilsson et al., 2009; Stevens & Au, 2021). The relationship between blood flow and blood pressure is linear as long as the vascular impedance is not altered. In this study the blood pressure was in focus as in previous in vivo studies where the vascular impedance has been altered, due to pharmaceutical intervention (Ahlgren et al., 2015; Ahlgren, Cinthio, Steen, et al., 2012) or physical activity (Ahlgren et al., 2023), it has been demonstrated that the correlation between blood pressure and antegrade longitudinal motion can be strong (Ahlgren et al., 2023; Ahlgren, Cinthio, Steen, et al., 2012) or very strong, with a correlation coefficient of up to 0.99 (Ahlgren et al., 2023).

A limitation of the present study is that the dimensions chosen for wall thickness and lumen were not physiological. These disparities mean that the results do not translate quantitatively to in vivo, but rather indicate some factors that might be of importance for the longitudinal motion. Other limitations were that the digital simulation model did not emulate the phantom models in every possible aspect and only simulate the increasing part of a pulse cycle. However, the digital simulation model did emulate the phantom model in the most important aspects for the study. The digital model consisted of two wall parts: one thinner part surrounded by a larger part. The friction between the two parts was either low or varied. Furthermore, as we were interested in how the pulse pressure (the variation in dynamic pressure between two points in time) influence the longitudinal motion it is adequate to simulate the pulse pressure as a general static step between two points. The results of the simulations showed some boundary effects. The displacements of the nodes at the boundaries were constrained to mimic the connections of the phantoms. The constraints cause adjacent elements to compensate by bending and stretching. The boundary conditions do not significantly affect the results in the regions of interest. Comparison of the results obtained from the phantoms must be made with some restraint. The pressure conditions were not identical due to the difference in flow resistance. Moreover, the echoes tracked were in the same region but not in identical positions as that depended on arbitrarily deposited glass beads in the phantoms. The same glass beads were, however, tracked in each cine loop of each phantom. The position of each bead, both the distance from the tapering and from the lumen, was likely to affect the magnitude of the displacement.

In conclusion, the conducive effects of geometry, low intramural friction and pressure on the longitudinal wall motion in these experimental settings are clear. Although the models are not based on in situ measurements, the results suggest that these factors could be of importance when explaining the antegrade longitudinal wall motion also in large human arteries. We believe this is a crucial step toward phantoms and models that accurately reproduce the longitudinal motion observed in vivo. Further studies are needed to elucidate the different components of the motion before they can be combined in a complete physiological model.

## FUNDING INFORMATION

This study was supported by grants from the Swedish Foundation for International Cooperation in Research and Higher Education, Swedish Research Council, the Medical Faculty, Lund University, the Skåne County Council's Research and Development Foundation, and the Research Foundation Flanders.

## CONFLICT OF INTEREST STATEMENT

The authors have no conflicts of interest.

## ETHICAL APPROVAL

Figures 1 and 2 were created from data collected from our previous studies. These studies were approved by the Ethics Committee of Lund University. All subjects gave informed consent according to the Helsinki Declaration.

## Data Availability

The data that support the findings of this study are available from the corresponding author, [MC], upon request.
